# Optimization and Clinical Validation of Colorimetric Reverse Transcription Loop-Mediated Isothermal Amplification, a Fast, Highly Sensitive and Specific COVID-19 Molecular Diagnostic Tool That Is Robust to Detect SARS-CoV-2 Variants of Concern

**DOI:** 10.3389/fmicb.2021.713713

**Published:** 2021-11-18

**Authors:** Pedro A. Alves, Ellen G. de Oliveira, Ana Paula M. Franco-Luiz, Letícia T. Almeida, Amanda B. Gonçalves, Iara A. Borges, Flávia de S. Rocha, Raissa P. Rocha, Matheus F. Bezerra, Pâmella Miranda, Flávio D. Capanema, Henrique R. Martins, Gerald Weber, Santuza M. R. Teixeira, Gabriel Luz Wallau, Rubens L. do Monte-Neto

**Affiliations:** ^1^Instituto René Rachou, Fundação Oswaldo Cruz, Belo Horizonte, Brazil; ^2^Centro de Tecnologia em Vacinas, UFMG/Fiocruz, Belo Horizonte, Brazil; ^3^Departamento de Microbiologia, Instituto Aggeu Magalhães, Fundação Oswaldo Cruz, Recife, Brazil; ^4^Departamento de Física, Universidade Federal de Minas Gerais, Belo Horizonte, Brazil; ^5^Núcleo de Inovação Tecnológica, Fundação Hospitalar do Estado de Minas Gerais, Belo Horizonte, Brazil; ^6^Visuri Equipamentos e Serviços, Belo Horizonte, Brazil; ^7^Departamento de Engenharia Elétrica, Universidade Federal de Minas Gerais, Belo Horizonte, Brazil; ^8^Departamento de Entomologia e Núcleo de Bioinformática, Instituto Aggeu Magalhães, Fundação Oswaldo Cruz, Recife, Brazil

**Keywords:** COVID-19, RT-LAMP, SARS-CoV-2, molecular test, respiratory virus, diagnostic test

## Abstract

The coronavirus disease 2019 (COVID-19) pandemic unfolded due to the widespread severe acute respiratory syndrome coronavirus 2 (SARS-CoV-2) transmission reinforced the urgent need for affordable molecular diagnostic alternative methods for massive testing screening. We present the clinical validation of a pH-dependent colorimetric reverse transcription loop-mediated isothermal amplification (RT-LAMP) for SARS-CoV-2 detection. The method revealed a limit of detection of 19.3 ± 2.7 viral genomic copies/μL when using RNA extracted samples obtained from nasopharyngeal swabs collected in guanidine-containing viral transport medium. Typical RT-LAMP reactions were performed at 65°C for 30 min. When compared to reverse transcriptase–quantitative polymerase chain reaction (RT-qPCR), up to cycle-threshold (Ct) value 32, RT-LAMP presented 98% [95% confidence interval (CI) = 95.3–99.5%] sensitivity and 100% (95% CI = 94.5–100%) specificity for SARS-CoV-2 RNA detection targeting *E* and *N* genes. No cross-reactivity was detected when testing other non–SARS-CoV virus, confirming high specificity. The test is compatible with primary RNA extraction–free samples. We also demonstrated that colorimetric RT-LAMP can detect SARS-CoV-2 variants of concern and variants of interest, such as variants occurring in Brazil named gamma (P.1), zeta (P.2), delta (B.1.617.2), B.1.1.374, and B.1.1.371. The method meets point-of-care requirements and can be deployed in the field for high-throughput COVID-19 testing campaigns, especially in countries where COVID-19 testing efforts are far from ideal to tackle the pandemics. Although RT-qPCR is considered the gold standard for SARS-CoV-2 RNA detection, it requires expensive equipment, infrastructure, and highly trained personnel. In contrast, RT-LAMP emerges as an affordable, inexpensive, and simple alternative for SARS-CoV-2 molecular detection that can be applied to massive COVID-19 testing campaigns and save lives.

## Introduction

Emerging viral infections continue to pose a major threat to global public health. In the past decades, different viral emergencies have been reported including the severe acute respiratory syndrome coronavirus (SARS-CoV), H1N1 influenza, Middle East respiratory syndrome coronavirus, Ebola vírus, Zika virus, and most recently, the new coronavirus has been described, which cause coronavirus disease 2019 (COVID-19; Wang et al., 2020; Zhu et al., 2020). COVID-19’s etiologic agent is SARS-CoV-2, which belongs to the Coronaviridae family, *Betacoronavirus* genus (Gorbalenya et al., 2020; Rambaut et al., 2020). People with COVID-19 have a wide range of symptoms reported such as fever, cough, anosmia, ageusia, headache, fatigue, muscle or body aches, sore throat, and shortness of breath or difficulty breathing. Some of these symptoms help spread the virus; however, human-to-human transmission from infected individuals with no or mild symptoms has been extensively reported (Bai et al., 2020; Rothe et al., 2020). This outbreak has spread rapidly; as of September 2021, there were more than 230 million confirmed COVID-19 cases with more than 4.7 million deaths recorded worldwide^1^. Isolation and quarantine of infected individuals are essential to viral spread and community dissemination of airborne pathogens and require an accurate, fast, affordable, readily available tests for massive population testing. In contrast to antibody detection, which may take weeks after the onset of the infection, detection of viral RNA is the best way to confirm the acute infection phase, the most important phase for viral shedding, so that rationally managed social distancing and lockdown can be implemented (Long et al., 2020; Wang et al., 2020).

**FIGURE 1:**
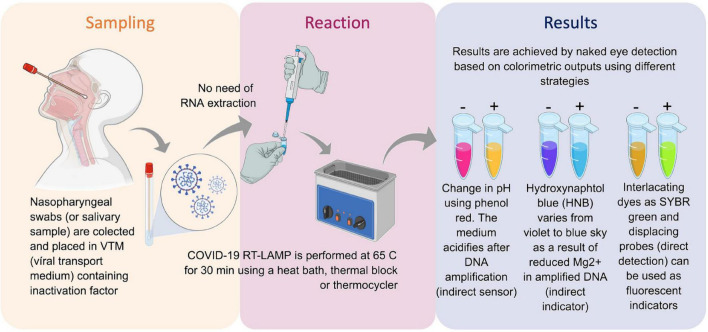
Reverse transcription loop-mediated isothermal amplification (RT-LAMP) for SARS-CoV-2 RNA detection and COVID-19 testing. Inactivated saliva samples or from nasopharyngeal swabs can processed for RNA extraction previously or be directly added to RT-LAMP reaction. Colorimetric output can be achieved by different sensors and can be read by naked eye. The whole procedure is rapid and simple and does not require complex infrastructures. Created with biorender.com.

Reverse transcriptase–quantitative polymerase chain reaction (RT-qPCR) is considered the gold-standard method for SARS-CoV-2 RNA detection, mainly targeting combinations of viral genome regions that codes for nucleocapsid protein (N), envelope protein (E), RNA-dependent RNA polymerase (RdRp), and other targets on the open reading frame (ORF1ab; Esbin et al., 2020). Although RT-qPCR assays have played an important role in the SARS-CoV-2 diagnosis, the technique has limitations for massive population testing such as processing time; it requires sophisticated equipment, infrastructure, and highly trained staff, as well as costly reagents with high demand and shortages around the world. Thus, developing complementary, inexpensive point-of-care (PoC) methods that are rapid and simple and allowing the use of alternative reagents for COVID-19 diagnosis test are urgently needed. Methods gathering these features can make affordable massive testing campaigns, including contact tracing strategies in highly dense countries, saving lives (Baek et al., 2020; Dudley et al., 2020; Song et al., 2021; Godfrey et al., 2020; Park et al., 2020; Wang, 2020; Yan et al., 2020; Yu et al., 2020; Anahtar et al., 2021). In this regard, reverse transcription loop-mediated isothermal amplification (RT-LAMP) has been shown to be an affordable technique applied to detect different pathogens (Mori and Notomi, 2009; Li et al., 2017). RT-LAMP has been used during Ebola outbreak (Kurosaki et al., 2016a,b) and for tracking Zika virus (da Silva et al., 2019) or *Wolbachia* (Gonçalves et al., 2014) in Brazilian mosquitoes. The method relies on specific DNA amplification at constant temperature without the need for sophisticated thermal cyclers (Zhang et al., 2020a). The amplified products can be visually detected through magnesium pyrophosphate precipitation, fluorescence emission from DNA intercalating dyes, agarose gel electrophoresis, lateral flow immunochromatography, magnesium chelating color indicators (Bhadra et al., 2021), and pH-dependent colorimetric reaction that changes from fuchsia (pink) to yellow (positive result) due to proton release during nucleic acid amplification (Tanner et al., 2015; Figure 1). The possibility of accessing results by the naked eye made RT-LAMP an exciting alternative that facilitates the use of COVID-19 molecular testing. Simple, scalable, cost-effective RT-LAMP–based alternatives for SARS-CoV-2 detection have emerged during pandemics including protocols for viral inactivation, quick run, RNA extraction–free and LAMP-associated CRISPR/Cas strategies (Baek et al., 2020; Broughton et al., 2020; Chow et al., 2020; Dudley et al., 2020; Song et al., 2021; Godfrey et al., 2020; Joung et al., 2020; L’Helgouach et al., 2020; Park et al., 2020; Rabe and Cepko, 2020; Bektaş et al., 2021; Bokelmann et al., 2021). On April 14, 2020, the RT-LAMP received the emergency use authorization from the United States Food and Drug Administration (FDA) for SARS-CoV-2 detection in COVID-19 diagnostics.

In this study, we optimized and validated a colorimetric RT-LAMP assay to detect SARS-CoV-2 RNA in clinical samples collected in different parts of Brazil, including samples with known SARS-CoV-2 variants of interest (VOIs) and concern (VOCs). After testing different primer sets for SARS-CoV-2 RNA detection by RT-LAMP, best results were achieved when using *N* gene or *N/E* genes-based strategies. 367 nasopharyngeal swabs collected in a guanidine-containing viral transport medium (VTM; Faria et al., 2021) from suspect patients were tested. The clinical validation revealed a sensitivity of 98% [95% confidence interval (CI) = 95.3–99.5%] with samples of cycle-threshold (Ct) values ranging from 15 to 32 with 100% specificity. We also demonstrated that RT-LAMP is affordable for the detection of more transmissible SARS-CoV-2 variants encompassing a number of genomic nucleotide changes. Part of the results presented here is the research basis of OmniLAMP^®^ SARS-CoV-2 kit, which was approved by the Brazilian Heath Regulatory Agency for COVID-19 molecular testing (Anvisa no: 10009010368) as an alternative for massive decentralized diagnostic in Brazil, which records the third-highest number of COVID-19 cases worldwide (see text footnote 1). Together with vaccination, RT-LAMP for COVID-19 diagnosis could help to improve better life quality during the pandemic, offering an alternative molecular testing for monitoring lockdown measures; traveling restrictions; the return of universities, schools, kindergartens; and sport league activities with worldwide impact.

## Results

### Reverse Transcription Loop-Mediated Isothermal Amplification Targeting SARS-CoV-2 *N*/*E* Genes Can Detect as Low as 19 Viral Copies/μL

**FIGURE 2:**
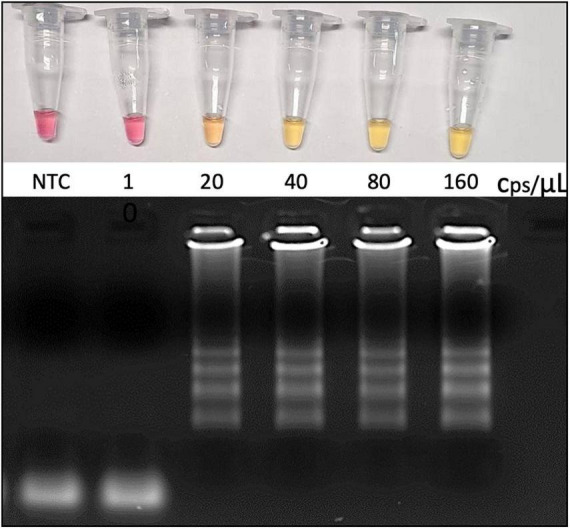
Analytical sensitivity as revealed by the limit of detection (LoD). RNA was extracted from VTM-nasopharyngeal swab, and the genome viral copies input was calculated based on SARS-CoV-2 *E* gene-harboring plasmid (Bioclin #K228-1) calibration curve. RT-LAMP reaction was performed at 65°C during 30 min using WarmStart^®^ colorimetric master LAMP mix (NEB #M1800) in 20 μL final volume (upper panel). Amplicons were resolved in 2% agarose gel and stained with GelRed^®^ (Biotium #41003) to confirm DNA amplification (bottom panel). cps/μL, viral genome copies per microliter; NTC, nontemplate control; VTM, viral transport medium (Bioclin #G092-1).

In order to access absolute analytical sensitivity of the colorimetric RT-LAMP for SARS-CoV-2 detection, we calculated the limit of detection (LoD), which is the lowest detectable concentration of viral nucleic acid, here represented in viral copies per microliter (/μL), which was determined based on a calibration curve from a known copy number load standard *E* gene-harboring plasmid. Purified SARS-CoV-2, obtained from infected Vero E6 cells, revealed an LoD equivalent to 0.44 ± 0.2 copies/μL, whereas RNA obtained from clinical samples (nasopharyngeal swab in VTM) resulted in an LoD of 19.3 ± 2.7 copies/μL. Validation was performed using clinical samples, confirming the LoD by colorimetric RT-LAMP, as well as by the visualization of the amplified DNA in agarose gel (Figure 2).

### SARS-CoV-2 Detection by Reverse Transcription Loop-Mediated Isothermal Amplification on Clinical Samples Presents 100% Specificity, Whereas Sensitivity Varies From 100 to 84%, Depending on the Viral Load

**FIGURE 3:**
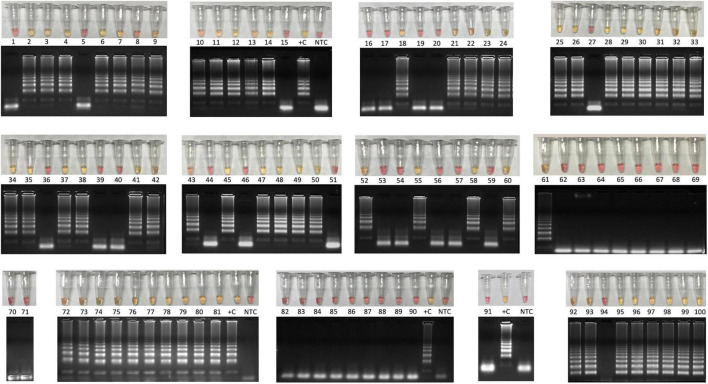
Colorimetric RT-LAMP for COVID-19 diagnosis validation using 100 clinical samples. Clinical samples were collected from symptomatic and hospitalized patients by nasopharyngeal swabs in partnership with CT-Vacinas/UFMG, Belo Horizonte, Brazil. Samples were obtained from different parts including Brazilian Southeast and Northeast regions. The reaction was performed at 65°C during 30 min using WarmStart^®^ colorimetric LAMP master mix (NEB #M1800) in 20 μL final volume. The RT-LAMP reaction targeted SARS-CoV-2 *N* gene. Yellow content indicates positive reaction, whereas the pink pattern reveals nonreagent samples. Amplicons were resolved in 2% agarose gel and stained with GelRed^®^ (Biotium #41003) to confirm DNA amplification. Latter pattern confirmed specific SARS-CoV-2 amplification that matches with yellow output tubes, which is not observed in pink nonreagent tests. +C, positive control using RNA extracted from laboratory-Vero E6 cultured inactivated SARS-CoV-2; NTC, nontemplate control. Clinimetric parameters from these samples are presented in Supplementary Figure S1.

The diagnostic accuracy for RT-LAMP was compared to the “gold-standard” technique RT-qPCR. The relative sensitivity was accessed in a panel of 367 clinical specimens from nasopharyngeal swab collected in VTM, including 254 positive and 113 negative samples according to the colorimetric RT-LAMP output that were previously characterized by RT-qPCR (Table 1). The colorimetric output was correlated with the visualization of amplified DNA after agarose gel electrophoresis (Figure 3).

**TABLE 1:** Estimated values comparing clinimetric parameters between colorimetric RT-LAMP and RT-qPCR on the detection of SARS-CoV-2 for molecular diagnosis of COVID-19.

RT-qPCR	Colorimetric RT-LAMP		Metrics % (95% CI)				
Ct value	Positive	Negative	Sensitivity	Specificity	Accuracy	PPV	NPV
15–30	171	0	100 (98–100)	100 (94.5–100)	100	100	100
15–32	199	4	98 (95–99.5)	100 (94.5–100)	99.95	100	99.95 (99.8–100)
15–34	221	13	94 (90.7–97)	100 (94.5–100)	99.90	100	99.9 (99.7–100)
15–36	245	29	89 (85.1–93)	100 (94.5–100)	99.74	100	99.7 (99.6–99.8)
15–40	254	48	84 (79.4–88)	100 (94.5–100)	99.60	100	99.6 (99.5–99.7)
Negative	0	65					

*Sensitivity: probability that the test result will be positive when the disease is present (true positive rate) = true positive/(true positives + false negatives); Specificity: probability that a test result will be negative when the disease is not present (true-negative rate) = true negatives/(true negatives + false positives); accuracy, PPV, and NPV depending on COVID-19 disease prevalence that was considered here as 2.5% according to the average value of two surveys during May and June 2020 (Hallal et al., 2020). PPV is the probability that the disease is present when the test is positive, whereas NPV is the probability that the disease is not present when the test is negative, and both are calculated as follows: PPV = sensitivity × prevalence/sensitivity × prevalence + (1 – specificity) × (1 – prevalence); NPV = specificity × (1 – prevalence)/(1 – sensitivity) × prevalence + specificity × (1 – prevalence); accuracy is the overall probability that a patient is correctly classified and is calculated as follows: =sensitivity × prevalence + specificity × (1 – prevalence). All calculations were performed using MedCalc (https://www.medcalc.org/) and VassarStats—Clinical Research Calculators (http://vassarstats.net/).*

**FIGURE 4:**
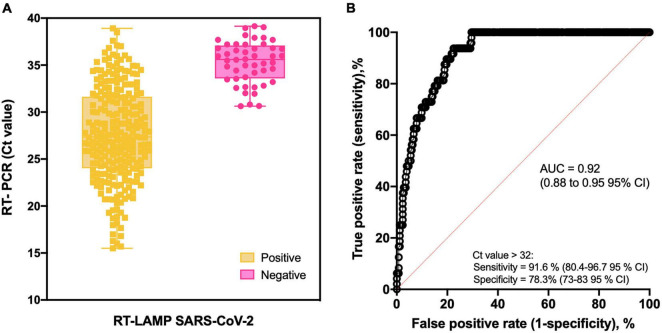
Colorimetric RT-LAMP for SARS-CoV-2 RNA detection. **(A)** Box-and-whisker representation of colorimetric RT-LAMP SARS-CoV-2–positive and –negative output (*x* axis) plotted in function of their respective RT-PCR Ct values (*y* axis). Forty-eight false negative samples were detected on RT-LAMP after Ct 32 despite other 55 being positive from Cts ranging from 32 to 39. **(B)** Receiver operating characteristic (ROC) curve constructed based on data presented in **A**. As summarized in Table 1, high-sensitivity values were obtained at the predicted cutoff.

The overall accuracy of colorimetric RT-LAMP compared to RT-qPCR was 99%, considering Ct values ranging from 15 to 40, with relative sensitivity of 84% (95% CI = 79.4–88%) and 100% (95% CI = 94.5–100%) specificity (Table 1). However, considering samples with equivalent RT-qPCR Ct value ≤ 32, RT-LAMP sensitivity is 98% (95% CI = 95.3–99.5%) and reaches 100% (95% CI = 94.5–100%) in samples with Ct value ≤ 30, whereas specificity is always 100% (Table 1), which means there are no false-positive hits. It is noteworthy that Ct > 32 RT-LAMP starts to present false-negative outputs (Table 1 and Figure 4); however, 55 samples were detected as positive on RT-LAMP with RT-qPCR Ct values ranging from 32 to 39 (Figure 4A). Receiver operating characteristic curve confirmed high sensitivity at RT-PCR equivalent Ct value > 32 for RT-LAMP on COVID-19 diagnostics (Figure 4B). The aforementioned results were achieved when using a multiplexed set of primers targeting *E* and *N* genes combined. However, prior to this, we performed the evaluation of N gene alone in 100 clinical samples (60 positive and 40 negative results) derived from hospitalized patients (Supplementary Figure 1). In this set of samples, we were also able to validate high sensitivity/specificity, absence of cross-reactivity with non–SARS-CoV viruses, and the capacity of SARS-CoV-2 variant detection (Supplementary Figure 1).

### Reverse Transcription Loop-Mediated Isothermal Amplification Targeting SARS-CoV-2 Does Not Cross-React With Other Viruses, Including Respiratory Ones

**FIGURE 5:**
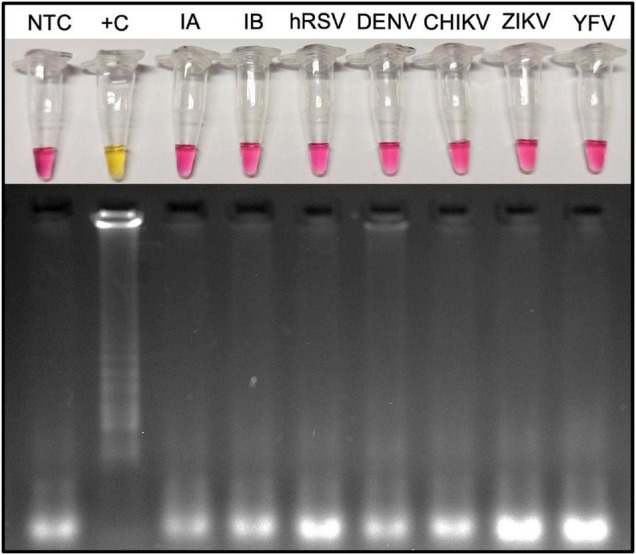
Microbial cross-reactivity assay to test SARS-CoV-2 RT-LAMP analytical sensitivity. The test was performed using potentially cross-reacting respiratory viruses or local occurring arboviruses. RT-LAMP reaction was performed at 65°C during 30 min, with additional 10 min, to confirm the absence of cross-reactivity when targeting SARS-CoV-2 *E* and *N* genes. The assay was performed using the WarmStart^®^ colorimetric LAMP 2× master mix (NEB #M1800). Yellow (positive) reaction is observed only when the template is SARS-CoV-2 viral RNA. hRSV, human respiratory syncytial virus; NTC, nontemplate control; M, molecular size marker. RT-LAMP amplification products were resolved in 2% agarose gel and stained with GelRed^®^ (Biotium #41003) to confirm DNA amplification. DENV3, dengue virus serotype 3; ZIKV, Zika virus; CHIKV, Chikungunya virus; YFV, yellow fever virus; Influenza A (H1N1/H3N2); and influenza B (Yamagata/Victoria).

The analytical specificity was confirmed by performing RT-LAMP for SARS-CoV-2 on putative cross-reacting viruses such as pathogens that colonize the human upper respiratory tract or that are associated with seasonal outbreaks in Brazil. None of the tested viruses [human influenza A virus/H1N1, influenza B virus, human respiratory syncytial virus (hRSV), dengue, Zika, Chikungunya, and yellow fever viruses] presented cross-reactivity on RT-LAMP using *E* an *N* gene as SARS-CoV-2 target (Figure 5). Similar results were obtained when using *N* gene alone as target (Supplementary Figure 1E). It reinforces the high specificity observed on clinical validation with no false-positive results (Figure 3). Thermodynamic and alignment analyses were performed on SARS-CoV-2 *N*, *E*, and *RdRp* RT-LAMP primer sets, revealing that there is no cross-reactivity over more than 300 non–SARS coronaviruses–derived genomes (Supplementary Table 1).

**FIGURE 6:**
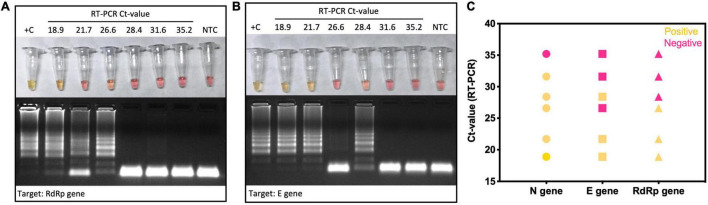
Colorimetric RT-LAMP for SARS-CoV-2 detection using genes *N*, *E*, and *RdRp* as target. Selected SARS-CoV-2–positive clinical samples by RT-qPCR were classified as low (Ct 18.9 and 21.7), medium (Ct 26.6 and 28.4), and high (Ct 31.6 and 35.2) Ct values for *E* gene. They were included as input for colorimetric RT-LAMP reaction using primers targeting *N*, *RdRp* **(A)**, and E genes **(B)**. RT-LAMP SARS-CoV-2 false-negative samples were more frequent when using *E* and *RdRp* genes as target **(C)**. RT-LAMP reaction was performed at 65°C during 30 min, using the WarmStart^®^ colorimetric LAMP 2× master mix (NEB #M1800). RT-LAMP amplification products were resolved in 2% agarose gel and stained with GelRed^®^ (Biotium #41003) to confirm DNA amplification. +C, positive control using SARS-CoV-2 RNA extracted from laboratory-cultured inactivated SARS-CoV-2; NTC, nontemplate control.

Six clinical samples previously confirmed as SARS-CoV-2 positive by RT-qPCR were subclassified as presenting low, medium, or high Ct values targeting *E* gene. All of them were tested by colorimetric RT-LAMP in independent reactions to test the performance of *N*, *E*, and *RdRp* genes as target to detect SARS-CoV-2. The samples with low Ct values (18.9 and 21.7) were positive for all tested primer sets, whereas *E* and *RdRp* genes started to present false-negative results from medium (26.6 and 28.4) Ct values (Figure 6). It indicates that the SARS-CoV-2 *N* gene is a better target for colorimetric RT-LAMP, detecting viral RNA in samples with equivalent RT-qPCR Ct values > 30 (Figure 6).

Colorimetric RT-LAMP sensitivity depends on the set of LAMP primers that can vary even within the same target. When RT-LAMP was performed on low viral load samples (Ct value for *E* gene ranging from 31.8 to 36.2), the *N* gene_Set1 was able to identify 4 of 12 (33.3%) true-positive samples. In contrast, *N* gene_Set2 or primer multiplex strategy (*N* gene Set1/Set2) allowed the detection of 11 of 12 (91.6%) true-positive samples (Supplemental Table 2).

### Colorimetric Reverse Transcription Loop-Mediated Isothermal Amplification Can Be Performed on Clinical Samples Without RNA Extraction

**FIGURE 7:**
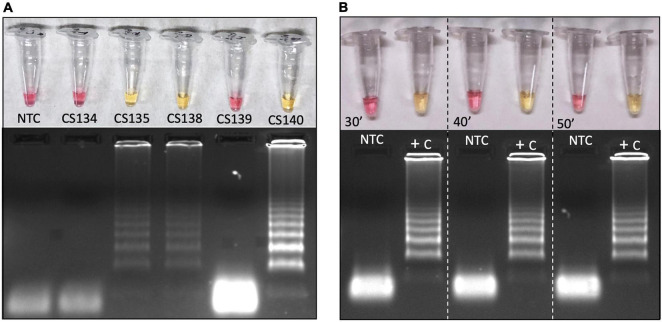
Colorimetric RT-LAMP to detect SAR-CoV-2 in RNA extraction–free clinical samples **(A)** or laboratory-cultured virus **(B)**. Clinical samples were derived from nasopharyngeal swabs placed on guanidine-containing viral transport medium, diluted 1:10. The RT-PCR Ct values for SARS-CoV-2 based on *E* gene are as follows: CS134 = 31.8, CS135 = 15.3, CS138 = 18.4, CS139 = 21.7, and CS140 = 24.6. RT-LAMP reaction was performed in 20 μL final volume, incubated at 65°C during 30, 40, or 50 min (inactivated virus) using WarmStart^®^ colorimetric LAMP master mix (NEB #M1800). Both clinical samples and viruses are RNA extraction–free samples. The amplification products (amplicons) were migrated in agarose gel at 2% to confirm amplification, as indicated by the characteristic ladder highlighted by GelRed^®^ staining. NTC, nontemplate control; CS, clinical sample; and +C, positive control.

Reverse transcription loop-mediated isothermal amplification performed in clinical samples, without any chemical or physical pretreatment or RNA extraction, returned positive output color in three of five samples (Figure 7A). In this assay, we used laboratory-cultured and inactivated SARS-CoV-2 and clinical samples without previous RNA extraction, showing that it is possible to use direct patients’ samples without preprocessing (Figure 7A). However, this should be taken with caution, as crude clinical samples may contain interferents that can block RT-LAMP reaction. Previous heat inactivation can be used to reduce this possibility. Here, only 1 μL of 1:10 solution of hydrochloride guanidine-containing VTM from nasopharyngeal swabs was added as a template to the SARS-CoV-2 LAMP reaction. Further analyses are being performed to establish the method sensitivity and feasibility for massive patient screening. All five samples had previous RNA extraction, for RT-PCR analysis, supporting that extraction process can increase detection sensitivity. We also tested the incubation time at 65°C reaction temperature. All SARS-CoV-2 control samples turned reaction color from fuchsia to yellow as indicative of DNA amplification, confirming positive reaction from the earliest time point tested (Figure 7B). In all tested intervals nontemplate controls were pink/fuchsia (negative) as expected, without any spurious late amplification, as confirmed by agarose gel electrophoresis showing no amplification bands on it (Figure 7B).

### Colorimetric Reverse Transcription Loop-Mediated Isothermal Amplification Allows the Detection of New SARS-CoV-2 Variants of Interests and Variants of Concern

**FIGURE 8:**
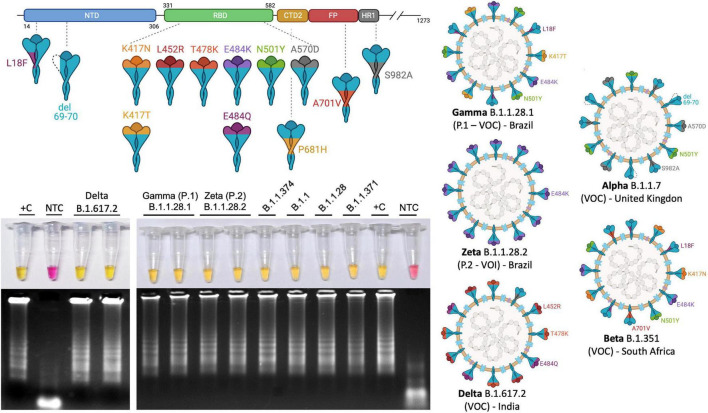
Colorimetric RT-LAMP allows the detection of SARS-CoV-2 VOCs and VOIs. RT-LAMP reaction was performed at 65°C for 30 min, using the WarmStart^®^ colorimetric LAMP 2× master mix (NEB #M1804), using multiplex *N*2/*E*1 primer sets. The amplicons were migrated in agarose gel at 2% to confirm amplification, as indicated by the characteristic ladder highlighted by GelRed^®^ staining. NTC, nontemplate control; CS, clinical sample; and +C, positive control. The top panel shows a schematic representation of SARS-CoV-2 spike protein (upper) and where the main mutations are highlighted and represented in SARS-CoV-2 virions (right hand side) present in VOC gamma (B.1), delta (B.1.167.2), and VOI zeta (P.2). The VOCs alpha (B.1.1.7) and beta (B.1.3.51), first reported in the United Kingdom and South Africa, respectively, are also represented. K417N: lysine-to-asparagine substitution at position 417 of spike protein at the receptor biding domain (RBD); V445A: valine-to-alanine substitution at position 445 and so on. L, leucine; Q, glutamine; E, glutamic acid; Y, tyrosine; T, threonine; P, proline; H, histidine; D, aspartic acid; S, serine; F, phenylalanine. del, deletion. Segments of SARS-CoV-2 protein NTD, N-terminal domain; CTD2, C-terminal domain 2 or C terminus of S1 fragment after furin cleavage; FP, fusion peptide; HR1, heptad repeat region 1. SARS-CoV-2 variants were previously sequenced. Variants of interest B.1.1.371 and B.1.1.374 were first reported in Saudi Arabia and Finland, respectively, (https://cov-lineages.org/). Created with biorender.com.

As a worldwide concern, SARS-CoV-2 VOI and VOC molecular detection could fail when applying S region–based RT-qPCR diagnostic methods due to mutations that would prevent primer annealing. In order to provide experimental evidences that RT-LAMP is a powerful molecular tool for detecting SARS-CoV-2 RNA, including VOCs and VOIs, we performed the tests on clinical samples that were previously identified as VOCs/VOIs by complete genome sequencing. All tested variants, including gamma (P.1 or B.1.1.28.1) and zeta (P.2 or B.1.1.28.2), originally reported in Brazil, and delta (B.1.167.2), first detected in India, were detected in colorimetric SARS-CoV-2 RT-LAMP (Figure 8), either by *N* gene alone as target (Supplementary Figure 1D) or by multiplex strategy using *N*2/*E*1 primer set, indicating that none of the mutant polymorphisms prevent specific primer annealing on RT-LAMP COVID-19 diagnosis (Figure 8).

## Discussion

The COVID-19 pandemics demanded a rapid global response in massive diagnostic solution to face the worldwide crisis. In this context, the RT-qPCR—considered the gold-standard technique for SARS-CoV-2 RNA detection—requires high-cost equipment, trained staff, and specialized laboratory infrastructure. In addition, during the COVID-19 pandemic, several health care centers and private laboratories competed for RT-qPCR kits and related products to meet the high diagnostic demand. In order to overcome the lacking of molecular testing and provide affordable alternatives, RT-LAMP had become one of the main hopes. Because of its simplicity, accuracy comparable with RT-qPCR to detect SARS-CoV-2 RNA, the fact that it does not require PCR machine, and for offering a naked eye readable colorimetric output, RT-LAMP is the focus of massive testing campaigns (Chow et al., 2020; Dudley et al., 2020; Song et al., 2021). This screening strategy is compatible with home, primary care clinics, point of entry (borders), schools, universities, sport leagues, and companies and can help to achieve a safe back-to-work and quarantine monitoring (Chow et al., 2020; Dudley et al., 2020; Song et al., 2021; Godfrey et al., 2020; Bokelmann et al., 2021). Since April 14, 2020, the United States FDA issued the emergency use authorization of Color SARS-CoV-2 RT-LAMP Diagnostic Assay from Color Health, Inc. (EUA no. EUA200539).

In order to provide an affordable SARS-CoV-2 detection tool, we validate a colorimetric RT-LAMP for the COVID-19 diagnosis using clinical samples collected from different parts of Brazil. The country has a flawed screening performance, testing fewer than 220 individuals per 1,000 people (May 2021)^2^ where the majority of tests rely on antibodies-based rapid tests, which are not the most reliable and recommended for mass screening and decision making to control local outbreaks. The test sensitivity of RT-LAMP is comparable to the gold standard RT-qPCR and clearly relies on the target choice, incubation time, viral load (asymptomatic patients, days of symptoms, and correct sampling), output reading, sample integrity, and quality (viral transport media, sample storage condition, preanalytical treatments, extraction procedure, and crude RNA extraction–free samples), and sample type (nasal, nasopharyngeal, saliva, sputum, and gargle lavage; Table 2).

**TABLE 2:** Comparison of SARS-CoV-2 RT-LAMP solutions, including key parameters on clinical validation.

Commercial name or acronym	Sample source	Transport medium	Target	Internal control	RNA extraction	Kit/output	Program	Sensitivity/specificity	LoD	Clinical sample tested	Local	References
OmniLAMP	Nasopharyngeal	VTM	*N, E* and *RdRp* genes	NA	Yes	NEB #M1800; #M1804 Color	65°C/30 min	100%/100% Up to RT-qPCR Ct value 30	20 copies/μL using clinical samples	467	CT-Vacinas-Fiocruz/UFMG, Belo Horizonte, Brazil	This study
	Nasopharyngeal	Saline	*N* gene and ORF1a	Human actin B gene	No	NEB #M1800 Color	65°C/30 min	87.5%/100%	25 copies/μL	62	Massachusetts General Hospital, Boston, MA, United States	Anahtar et al., 2021
					Yes			90%/100%		40		
	NI	NI	*N* gene; ORF1ab	NI	Yes	NEB #M1800 Color	65°C/30 min	100%/100%	240 copies/Rx	62	Paraná Central Laboratory, Curitiba, Brazil	Aoki et al., 2021
	Saliva	NA	*N* gene	NI	No, heated	NI	63°C/30 min	78.9%/100%	NI	244	Sírio-Libanês Hospital, São Paulo, Brazil	Asprino et al., 2020
	Nasal	NI	*N* gene	NI	Yes	NEB #M1800 Color	65°C/30 min	NI	10^–7^ (equivalent to Ct 34 in RT-PCR)	14	National Medical Center, Republic of Korea	Baek et al., 2020
ALERT	Nasal, saliva	PBS	*N* gene	BPIFA1 gene	Yes or without extraction (lysate samples)	NEB *Bst* 3.0 and RTx; #M1800 Fluorescence	63°C/45 min	95%/97%–100%	2 copies/μL	47	Hôpital Saint Louis, Paris, France; Pontifica Universidad Católica, Santiago, Chile	Bektaş et al., 2021
	Throat and nasopharyngeal	UTM	*N* gene;	NI	No (lysate samples)	NEB #M1800	65°C/30–40 min	71.15%/96.77% (30 min) 76,9%/96/77% (35 or 40 min)	NI	180	Rambam Health Care Campus, Haifa, Israel	Ben-Assa et al., 2020
LAMP-OSD	Nasopharyngeal and oropharyngeal and SARS-CoV-2 spiked saliva		*N* gene; *ORF1ab* (*NSP3* and *RdRp* genes)	NI	Yes	NEB *Bst* 2.0 polymerase; WarmStart RTx + betaine and additional MgCl_2_ + FAM Fluorescence	65°C/90 min	NI	10 copies/Rx	NA	NA	Bhadra et al., 2021
Cap-iLAMP	Gargle lavage	NA	*N* gene; *ORF1ab*		No, heated samples	NEB #M1800 Color + SYTO9 Fluorescence	65°C/25–30 min	97,1%/	500 copies/Rx	192	NI	Bokelmann et al., 2021
	Nasopharyngeal and oropharyngeal	BD UVTM	*N* gene and *E* gene	Human actin B gene	Yes	NEB #M1800 Color + QuantiFluor (Fluorescence)	65°C/30–40 min	95.6%/99.2%	8 copies/μL	857	New York Presbyterian Hospital Weill Cornell Medical Center, NY, United States	Butler et al., 2020
COVID-19-LAMP	Nasopharyngeal, sputum, and throat		*ORF3a*, *E* gene	NI	Yes	NEB colorimetric WarmStart	63°C/60–90 min	98.2%/100%	42 copies/Rx	223	University of Hong Kong Hospital, China	Chow et al., 2020
	Nasal and oral	PBS	*ORF1a*	human 18S RNA	Yes	NEB colorimetric WarmStart	63°C/30 min	93.8%/90.4%	100 copies/Rx up to Ct 35	466	Erasto Gaertner Hospital, Curitiba, PR, Brazil	de Oliveira Coelho et al., 2021
	Nasopharyngeal	SPS	*N* gene and *ORF1ab*	NI	Yes	NEB *Bst* 2.0, 3.0, RTx WarmStart +EvaGreen Color and Fluorescence		8.3%–100%/100%	200 copies/Rx		University Hospital of Salamanca, Spain	García-Bernalt Diego et al., 2021
	Nasopharyngeal	UTM, VTM or PBS	*ORF1a*	NI	No	NEB #E1700 Fluorescence	63°C/40 min	81%/100%	62.5 copies/μL	137	University of Wisconsin – Madison Hospital and Clinics, United States	Dudley et al., 2020
Penn-RAMP RPA+ LAMP	Nasal (spiked samples)	NA	*ORF1ab*	NI	NA	OptiGene Isothermal Mastermix (ISO-001) + EvaGreen dye Loopamp 2019-SARS-CoV-2 Detection Reagent Kit (Eiken Chemical, Tokyo, Japan) + Leuco Crystal Violet	63°C/50 min	100%/NI	7 copies/Rx	NA	NA	Song et al., 2021
	Saliva; throat and nasal	VTM	*N* gene	NI	No, chelating agent treatment	NEB Color	65°C/30 min	90%100%	10^5^ copies/mL	62	Rambam Health Care Campus in Haifa, Israel	Flynn et al., 2020
OptiGene COVID-19 RT-LAMP	Nasopharyngeal	VTM	*ORF1a*	NI	Yes, also tested without RNA extraction	OptiGene GspSSD 2.0 Opti-RT Fluorescence	65°C/20 min	97%/99%	100–200 copies/Rx	196	Hampshire Hospitals NHS Foundation Trust, United Kingdom	Fowler et al., 2021
	Nasopharyngeal	VTM	*ORF1a*, *ORF8*, *S* and *N* genes		No	NEB Bst 2.0 +EvaGreen Fluorescence	65°C/60 min	100%/100%	50 copies/μL	20	OSF Healthcare. Peoria, IL, United States	Ganguli et al., 2020
	Nasopharyngeal	NI	*N* gene	NI	Yes	NEB #M1800 Color +EvaGreen Fluorescence	65°C/50 min	NI	625 copies/Rx	14	Hospital Alfa Medical Center, Guadalupe, México	González-González et al., 2021
	Throat	VTM	*ORF1ab*, *S* gene and *N* gene	Human actin B gene	Yes	NEB #M1800 Color	65°C/30 min	NI	2 copies/25 μL	16	Shenzhen Luohu People’s Hospital in China.	Huang et al., 2020
	Nasopharyngeal	BD UVTM	NI	NI	Yes	SARS-CoV-2 detection kit (Eiken Chemical Co.) Turbidimetry Fluorescence	62.5°C/35 min	56.6%/98.4%	6.7 copies/Rx	124	University Hospital, Japan	Inaba et al., 2021
	Nasopharyngeal	NI	NI	NI	Yes	Loopamp 2019-SARS-CoV-2 Detection Reagent Kit (Eiken Chemical, Tokyo, Japan) Turbidity	62.5°C/35 min	100%/97.6%	101 copies/μL	76	National Institute of Infectious Diseases, Japan	Kitagawa et al., 2020
EasyCOV	Saliva	VTM	NI	NI	No	NEB E1700 + 1 M betaine/fluorescence	65°C/30 min	72.7%/95.7%	Equivalent to Ct 35 in RT-PCR	123	Montpellier University Hospital, France	L’Helgouach et al., 2020
	Saliva	Saline	*N*-A gene	NI	No, heat + Prot. K lysis	NEB #M1800 Color	62.5°C/30–60 min	NI	< 10 copies/μL (200 copies/Rx)	5	Washington University School of Medicine; Barnes-Jewish Hospital; the Institute of Clinical and Translational Sciences; Tissue Procurement Core, United States	Lalli et al., 2021
	Throat	NI	*N* gene	NI	Yes	NEB *Bst* 3.0; WarmStart RTx; Q5 HF DNA polymerase Color or fluorescence	62.5°C/30–40 min	Sensitivity was 100% for 393 copies/Rx; 80% for 79 copies/Rx and 60% for 16 copies/Rx	118.6 copies/25 μL or 4.7 copies/μL	56	Nantong Third Hospital, China	Lu et al., 2020
	Nasopharyngeal and throat	NI	*RdRp*	NI	Yes	NEB #M1800 Color	65°C/60 min	95.74%/99.95%	25 copies/Rx	2,120	Ramathibodi Hospital, Mahidol University, Bangkok, Thailand	Nawattanapaiboon et al., 2021
	Nasopharyngeal	VTM	*ORF1ab*	NI	Yes, magnetic bead extraction	MicrosensDx RapiPrep	65°C/25 min	80%/100%	Not determined	21	National Health Service Care Home, United Kingdom	Österdahl et al., 2020
	SARS-CoV-2 isolated from MRC-5 infected cells	NA	*NSP3* gene (*ORF1ab*) *S* gene; *N* gene	NI	Yes	NEB *Bst* 3.0; + SuperScript IV RT Invitrogen + or leuko–crystal violet Color NEB #M1800 + SYTO-9 Fluorescence	69°C or 65°C/30 or 60 min	NI	100 copies/Rx or 10^–6^ RNA dilution	NA	NA	Park et al., 2020
	Saliva, nasal and nasopharyngeal	Saline	*ORF1ab* (*As1/1e*); *ORF1a-C* and *N* gene	NI	No	NEB #M1800 and #E1700 Color and fluorescence	65°C/30–60 min	NI	1 copy/μL	NA	NA	Rabe and Cepko, 2020
LAMP-BEAC	Nasopharyngeal and saliva	NI	*E*, *N* genes; *Orf1ab* (*As1/1e*);	Human statherin mRNA	No, TCEP/EDTA and heat treated	NEB #E1700 and labmade *Bst* FL Fluorescence	60°C–65°C/45 min	NI	More than 100 copies/μL	82	NA	Sherrill-Mix et al., 2021a
	Nasal and nasopharyngeal	Amies medium	*ORF1a* and *N* gene	NI	Yes	NEB #M1800 Color	65°C/30 min	100%/99.7% up to Ct 25	100 copies/Rx or 4 copies/μL	792	University Hospital Heidelberg and municipal COVID-19 testing station, Germany	Thi et al., 2020
		Dry swab			No, heat treated or directly included in LAMP reaction		65°C/30 min	90.5%/99.5% up to Ct 25 in RT-PCR (hot swab)		343		
								93.8%/94.1% up to Ct 25 in RT-LAMP (direct swab)		235		
One-pot RT-LAMP	NA	NA	*N* gene	NA	pUC57-N gene (synthetic)	NEB *Bst* 3.0 DNA/RNA polymerase + EvaGreen +Rox Fluorescence	59°C/50 min	NI	6 copies/μL	NA	NA	Wang, 2020
	Nasopharyngeal	VTM	*ORF1ab*	NI	No	NEB #M1800 Color	63°C/30 min	75%/100%	2.5 copies/uL Spiked samples on VTM	20	Columbia University Irving Medical Center	Wei et al., 2021
	Respiratory swabs and bronchoalveolar lavage fluid		*ORF1ab* and *S* gene	NI	Yes	Loopamp? 2019-SARS-CoV-2 Detection Reagent Kit (Eiken Chemical, Tokyo, Japan) Turbidity or fluorescence (+ calcein)	63°C/18–60 min	100%/100%	20–110 copies/Rx	130	PLA General Hospital, Beijing, China	Yan et al., 2020
iLACO	Respiratory (not detailed)	NI	*ORF1ab*	NI	Yes	NEB #M1800 Color	65°C/ ≥20 min	89.9%/NI	10 copies/μL (detection threshold of 60 copies/μL); equivalent to 35–37 Ct in RT-PCR	248	Shenyang province, China	Yu et al., 2020
	Respiratory swabs (not detailed)	VTM	*N* gene and *ORF1a*	NI	Yes	NEB #M1800 + SYTO-9 Color and fluorescence	65°C/30 min	NI	4.8 copies/μL	6	Wuhan Institute of Virology, China	Zhang et al., 2020a
	Synthetic SARS-CoV-2 RNA	NA	*N* gene; *E* gene and *As1e* gene (*ORF1a*)	Human actin B gene	Yes	NEB #M1800 + SYTO-9 Color and fluorescence	65°C/20 min	87.5%	2 copies/μL	NA	NA	Zhang et al., 2020b

*NEB #M1800, WarmStart Colorimetric RT-LAMP 2 × Master Mix; NEB #E1700, WarmStart LAMP kit (DNA and RNA); RdRp, RNA-dependent RNA polymerase (harbored by ORF1ab SARS-COV-2 genome region); NI, noninformed; NA, not applied; UTM, Universal Transport medium; VTM, viral transport medium, commonly containing, Hank’s balanced salt solution at pH 7.4 containing BSA (1%), amphotericin (15 μg/mL), penicillin G (100 units/mL), and streptomycin (50 μg/mL); Prot K, proteinase K; BD UVTM, Becton–Dickinson Universal Viral Transport Media system; SPS, sample preservation solution; QuantiFluor, Promega system for dsDNA quantification using a DNA intercalating dye; Prot. K, proteinase K; Cps, copies; Rx, reaction; RdRp, RNA-dependent RNA polymerase; LoD, limit of detection; and BPIFA1, bactericidal/permeability-increasing fold-containing family A1.*

Upon RNA extraction from nasopharyngeal swab–derived clinical samples, we found an LoD of 20 viral genomic copies/μL, confirming previous studies based on *N* SARS-CoV-2 target (Anahtar et al., 2021; Bokelmann et al., 2021; González-González et al., 2021). It is worth noting that when using nonclinical SARS-CoV-2 extracted RNA or synthetic target, the LoD reaches less than 0.5 copies/μL. This can be explained by the presence of interferents such as VTM, host cells, and enzymes that could reduce the yield (Dudley et al., 2020; Nawattanapaiboon et al., 2021). In this regard, we have to be careful when interpreting LoD calculated using nonclinical samples. Nevertheless, extracted samples are rich enough in viral genomic copies to meet SARS-CoV-2 clinically relevant levels.

Clinical validation of RT-LAMP for COVID-19 diagnosis relies on calculating parameters, such as sensitivity, specificity, positive predictive value, negative predictive value, and accuracy compared to the gold standard RT-qPCR. We have to be careful when associating the RT-LAMP sensitivity, and indirect assumption on RT-qPCR viral load is not straightforward because of some technical concerns. It is well accepted that Ct values can be representative of viral load. However, this parameter could lead to misinterpretation when comparing different kits, targets, and nonstandardized samples. A survey conducted by the College of American Pathologists on more than 700 laboratories, reported a variation as much as 14 cycles among different methods on the same batch material. Single laboratories using different platforms and targets in SARS-CoV-2 molecular testing can represent a potential variability on Ct values (Rhoads et al., 2020). Considering previous convergent reports and presuming different targets and platforms, the data from the literature show that with an RT-qPCR Ct 30 cutoff, RT-LAMP sensitivity for SARS-CoV-2 detection is close to 100% (Schermer et al., 2020; Thi et al., 2020; de Oliveira Coelho et al., 2021; García-Bernalt Diego et al., 2021) and eventually with a higher threshold Ct 35 as well (L’Helgouach et al., 2020; de Oliveira Coelho et al., 2021). Indeed, we confirm that up to Ct 30 RT-LAMP returned 100% sensitivity for SARS-CoV-2 detection, reaching 98 and 94% when considering Ct values up to 32 and 34, respectively. Curiously, Kim et al. (2021) found that samples from hospitalized patients presenting Ct value of 28.4 or less were infective to human cell culture (Kim et al., 2021), an evidence based on *in vitro* extrapolation that RT-LAMP sensitivity is compatible with the threshold of infectivity. This reinforces that simple, robust, and reliable RT-LAMP meets clinical requirements, presenting similar COVID-19 diagnostic accuracy as RT-qPCR (Österdahl et al., 2020; Inaba et al., 2021; Jang et al., 2021).

**FIGURE 9:**
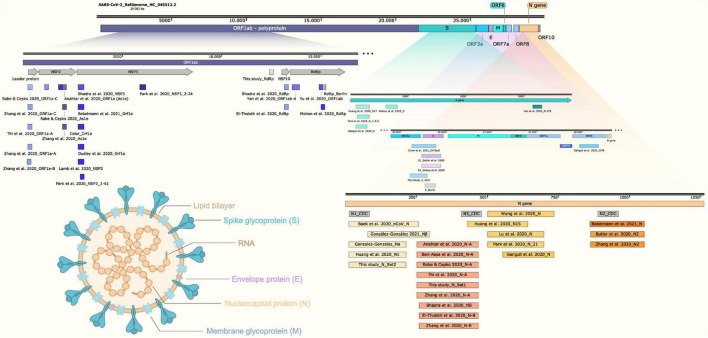
Schematic representation of SARS-CoV-2 genome indicating the amplicons for the COVID-19 molecular diagnostics by RT-LAMP. Structural representation of SARS-CoV-2 virion shows the main particle parts. LAMP primer regions are indicated as previously reported (Baek et al., 2020; Ben-Assa et al., 2020; Butler et al., 2020; Chow et al., 2020; Dudley et al., 2020; Song et al., 2021; Ganguli et al., 2020; Huang et al., 2020; Lamb et al., 2020; Lu et al., 2020; Mohon et al., 2020; Park et al., 2020; Rabe and Cepko, 2020; Thi et al., 2020; Yan et al., 2020; Yu et al., 2020; Zhang et al., 2020a,b; Anahtar et al., 2021; Bhadra et al., 2021; Bokelmann et al., 2021; González-González et al., 2021). ORF, open reading frame; RdRp, RNA-dependent RNA polymerase; NSP, nonstructural protein. Schematic representation created using Snap Gene Viewer software version 5.0.7; N1, N2, and N3_CDC correspond to the amplicons for SARS-CoV-2 detection by RT-PCR. Created with biorender.com.

The choice of SARS-CoV-2 genomic target plays an important role when selecting the RT-LAMP method for COVID-19 diagnosis. Several research groups have tested different regions on SARS-CoV-2 genome with the potential to generate RT-LAMP primers. Once the majority of primers were designed using the open source software Primer Explorer, it is expected that at some point, the default algorithm returned the same result or overlapping regions, independently identified in a context where molecular biology scientists everywhere in the world are working to tackle COVID-19 (Figure 9). According to our data compilation, *N* gene and *ORF1ab* regions (overlapping *NSP3*, *As1e*, and *RdRp*-coding sequences) were the most frequent targets chosen for SARS-CoV-2 RT-LAMP (Table 2 and Figure 9). Ganguli et al. (2020) and Zhang et al. (2020b) arrived at the same conclusion when selecting the SARS-CoV-2 *N* gene-targeting primer set after confirming better performances for RNA viral detection when compared to other targets (Ganguli et al., 2020; Zhang et al., 2020b). When testing *N*, *E*, and *RdRp* genes in true-positive, previously RT-qPCR–characterized clinical samples, we observed more false-negative outputs from assays using *E* and *RdRp* genes, corroborating what was previously reported. We also highlight that primer subsets within the same *N* target gene can contribute differentially to RT-LAMP test sensitivity (Supplementary Table 2). Furthermore, multiplexing different primer sets is encouraged in order to increase sensitivity (Figure 9; Kim et al., 2019; Mautner et al., 2020; Zhang et al., 2020b).

Another important (almost neglected) point is the fact that, although inspired by RT-qPCR target selection, few SARS-CoV-2 RT-LAMP approaches reported an internal control target to confirm the presence of human RNA and monitor sampling or extraction process (de Oliveira Coelho et al., 2021). Wilson-Davies et al. (2021) pointed out that the lack of amplification can happen for different reasons concerning the whole reaction, a specific well, or due to inhibitory substances, highlighting the importance of including internal control even before nucleic acid extraction, in order to be considered a reliable SARS-CoV-2 LAMP assay (Wilson-Davies et al., 2021). In this study, all clinical samples were previously characterized by RT-qPCR, including human RNAse P as housekeeping gene (internal control). In the current OmniLAMP^®^ assay, we included human b-actin RNA (rACTB) as internal control. Other constitutive targets for SARS-CoV-2 RT-LAMP include BPIFA1 (Bektaş et al., 2021), human 18S RNA (de Oliveira Coelho et al., 2021), and Statherin RNA (Sherrill-Mix et al., 2021a; Table 2).

Similar to its high sensitivity, obtained in this work and by other studies, the SARS-CoV-2 RT-LAMP specificity is undoubtedly high and is frequently reported as 100% without any cross-reactivity with other respiratory or SARS-CoV–unrelated viruses (Chow et al., 2020; Mohon et al., 2020; Park et al., 2020; Aoki et al., 2021; Nawattanapaiboon et al., 2021). We also confirm that the SARS-CoV-2 RT-LAMP solution presented here is highly specific and does not cross-react with Brazilian occurring seasonal influenza A and B, hRSV, or arboviruses.

Despite the advantages presented by purified and nucleic acid–enriched samples for SARS-CoV-2 RT-LAMP, RNA extraction–free protocols have attracted attention as they can be noninvasive (saliva-based), do not require additional steps and equipment, and fulfill point-of-sampling requirements. Indeed, the preanalytical phase on RT-LAMP is the bottleneck for PoC applications. For this reason, several studies highlighted the feasibility of primary RNA extraction–free approaches for SARS-CoV-2 RNA detection (Asprino et al., 2020; Ben-Assa et al., 2020; Dudley et al., 2020; Esbin et al., 2020; L’Helgouach et al., 2020; Schermer et al., 2020; Srivatsan et al., 2020; Anahtar et al., 2021; Lalli et al., 2021; Wei et al., 2021). Pretreatment of saliva samples includes heat sample inactivation, and the use of lysis/stabilizing buffers that can contain proteinase K, TCEP, EDTA, and DTT could help the viral RNA assessment maintaining its integrity (Ben-Assa et al., 2020; Lamb et al., 2020; L’Helgouach et al., 2020; Smyrlaki et al., 2020; Lalli et al., 2021; Newman et al., 2021; Yang et al., 2021). Caution must be taken when running colorimetric RT-LAMP as pretreatment could interfere on result outputs. One of the main limitations for direct sample test by colorimetric RT-LAMP based on pH-sensing is the false-positive result upon input sample addition (previous to amplification) because of naturally acidic samples (Thi et al., 2020; Bokelmann et al., 2021). To prevent spurious amplification due to the presence of DNA from oral microbiome, food, or host cells on primary samples, Bokelmann et al. (2021) treated samples with λ exonuclease that acts by preferentially digesting 5′-phosphorylated DNA, leaving nonphosphorylated primers or LAMP products intact (Bokelmann et al., 2021). Here we showed the preliminary results on RNA extraction–free (also pretreatment free) diluted 10× in hydrochloride guanidine-containing VTM nasopharyngeal samples directly accessed to compare colorimetric results. Three of five RT-qPCR true-positive, directly accessed samples returned positive yellow output on colorimetric RT-LAMP for SARS-CoV-2 detection. This provides clues on the use of unextracted samples for massive COVID-19 testing campaigns with a trade-off on cost-benefits for LoD and test sensitivity. A recent study on 559 swabs and 86,760 saliva samples performed a sample preparation method for RNA extraction–free and found diagnostic sensitivity of 70.35 and 84.62%, respectively, for swab and saliva samples (Kidd et al., 2021). Most of the high and medium viral load samples will be detected on unextracted protocols. However, to meet RT-qPCR detection sensitivity levels, this requires some type of purification step and RNA concentration (Broughton et al., 2020; Park et al., 2020; Zhang et al., 2020a).

We are currently observing rapid converging evolution of SARS-CoV-2 during the COVID-19 pandemic worldwide. Several reports alert for the emergence of VOIs and VOCs such as the alpha (B.1.1.7), first detected in England (ECDC threat assessment brief on December 20, 2020; European Centre for Disease Prevention and Control, 2020); Beta (B.1.351), initially reported from South Africa (Tegally et al., 2021); gamma (P.1 or B.1.1.28.1), which was identified in Japan but obtained from a traveler from Brazil (Faria et al., 2021); and more recently, the VOI kappa (B.1.617.1) and VOC delta (B.1.617.2) detected in India, responsible for the majority of new COVID-19 cases in many countries in different parts of the world. The regional selection of SARS-CoV-2 VOC is associated with higher transmissibility, mortality and reduced neutralizing antibody response (Shah et al., 2020; Davies et al., 2021a,b; Li et al., 2021). In Brazil, we observed the emergence of different SARS-CoV-2 VOCs and VOIs, including gamma (P.1), zeta (P.2; Resende et al., 2021a; Voloch et al., 2021), B.1.1.33.9 (N.9; Resende et al., 2021c), and B.1.1.33.10 (N.10; Resende et al., 2020, 2021b). A plethora of mutations is observed in these variants, including N501Y, E484K/Q, K417N/T, A570D, and the Δ69–70 at the SARS-CoV-2 S protein sequence, which was associated with detection failures by S-target RT-qPCR methods (Brown et al., 2021). For SARS-CoV-2 RT-LAMP detection, few studies selected S-coding protein region as a target (Figure 9). In addition, isothermal amplification for SARS-CoV-2 RNA detection strategies is commonly addressed as multiplex targeted, making RT-LAMP a good choice even for SARS-CoV-2 variant detection. Indeed, here we reported that singleplex *N* gene-based or multiplex *N2*/*E1*-based RT-LAMP was able to perfectly detect VOCs and VOIs circulating in Brazil such as gamma (P.1), zeta (P.2), B.1.1.374, and B.1.1.371 (Figure 8 and Supplementary Figure 1D), the two latter first detected in Finland and Saudi Arabia^3^. Recent efforts made by Sherrill-Mix et al. (2021a,b) showed a beacon-based RT-LAMP strategy designed to precisely identify alpha (B.1.1.7) SARS-CoV-2 variant (Sherrill-Mix et al., 2021a,b), a promising tool not only for massive screening but also to monitor VOC/VOI SARS-CoV-2 spreading.

The colorimetric RT-LAMP is a reliable molecular tool for detecting SARS-CoV-2, providing rapid and easy-to-read results, compatible with high-throughput screenings and PoC requirements. This test is especially important for nations with poor diagnostic conditions, such as Brazil, where RT-qPCR COVID-19 diagnostic is far from ideal to control disease spreading. The RT-LAMP sensitivity can be equivalent to those reported from the gold standard RT-qPCR method and also present 100% specificity. Results are commonly obtained after 30-min reaction and if needed, additional 20 min was not associated with spurious unspecific amplification. Sample collection in guanidine-containing VTM has been described as a useful strategy to avoid contamination of health care workers during sample manipulation. RT-LAMP primer selection can directly interfere on sensitivity, being *N* genes the best target for SARS-CoV-2 RNA detection with fewer false-negative results, especially in low viral load samples, which is improved upon multiplexing *E/N* targets. Colorimetric RT-LAMP is also compatible with detecting SARS-CoV-2 VOIs and VOCs, being robust to cope with the monitoring of emerging new SARS-CoV-2 variants and that can be easily adapted. We thus reinforce and recommend the use of RT-LAMP for massive testing as a decentralized PoC alternative to avoid SARS-CoV-2 spread and to tackle COVID-19.

## Materials and Methods

### Clinical Samples, Reverse Transcriptase–Quantitative Polymerase Chain Reaction, and Ethics Statement

In total, 467 clinical samples were included in this study. Initially, 100 nasopharyngeal clinical samples were obtained from hospitalized patients in different parts of Brazil from April to July 2020. The samples derived from this first batch were tested by RT-LAMP using *N* gene alone as target, and the group presented a median age of 60 years, and 60% of patients were male. An additional 367 samples were included in the study. They were obtained from symptomatic patients, considered COVID-19 suspected cases, during September until November 2020 in Belo Horizonte, Minas Gerais, Brazil. The samples from the latter group were validated by RT-LAMP targeting *E* and *N* genes combined and were characterized with a median age of 46 years old, in whom 75% of patients were female.

Nasopharyngeal swabs were collected and maintained in 2 mL VTM (Bioclin, Belo Horizonte, Brazil #G092-1) at room temperature until RNA extraction or direct dilution for LAMP reaction. The VTM contains guanidine chloride as inactivation agent and to preserve viral RNA. All procedures were performed inside a biosafety level 2 cabinet. RNA extraction was performed using the QIAamp^®^ Viral RNA Mini Kit (Qiagen #52906), following manufacturer instructions. The molecular diagnostic routine was performed by RT-qPCR using the SARS-CoV-2 commercial kits produced at Fundação Oswaldo Cruz [Kit Molecular SARS-CoV-2 E/RP, from Bio-Manguinhos/Fiocruz, based on Charité/Berlin protocol, and Kit Biomol OneStep/COVID-19 from IBMP/Fiocruz, based on China/Centers for Disease Control and Prevention (CDC) protocol with recommended targets polyprotein *ORF1ab* and *N* gene]. RT-qPCR was carried out using the 7500, ViiA 7 real-time PCR systems (Applied Biosystems, Foster City, CA, United States) or the dual-channel Open qPCR machine (Chai, Santa Clara, CA, United States), following the temperature program profile of 95°C for 3 min, followed by 40 cycles of amplification (95°C/15 s and 60°C/1 min). Influenza and hRSV samples were kindly provided by IOM/FUNED, and the arbovirus samples are part of the collection from the Laboratório de Imunologia de Doenças Virais at Oswaldo Cruz Foundation. All procedures involving human participants and collection and use of clinical samples and data were in accordance with ethical standards and approved by the local Research Ethics Committee involving human beings at Instituto René Rachou, Fundação Oswaldo Cruz, under license protocol no. 4084902 and CAAE (certificate of presentation for ethical appreciation): 31984720300005091. The ethics approval was issued on June 12, 2020. SARS-CoV-2 VOCs and VOIs included in this study were isolated from symptomatic patients (Ct value < 25, using *E* gene as target on RT-qPCR—Kit Molecular SARS-CoV-2 E/RP Bio-Manguinhos Fiocruz), in the State Pernambuco, Northeast Brazil (Bezerra et al., 2021; Supplementary Table 3). The study was approved by the local Human Research Ethics Committee (CAAE: 32333120.4.0000.5190). The genomes of SARS-CoV-2 VOI and VOCs generated are deposited on GISAID according to the following accession codes: EPI_ISL_2221860, EPI_ISL_2221850, EPI_ISL_2221873, EPI_ISL_2221890, EPI_ISL_2221902, EPI_ISL_2221885, EPI_ISL_2221844, and EPI_ISL_2221866.

### Reverse Transcription Loop-Mediated Isothermal Amplification Primer Design

Reverse transcription loop-mediated isothermal amplification primers were designed based on SARS-CoV-2 reference genome (GenBank accession NC_045512.2) using the open source software Primer Explorer V5^4^ or the New England Biolabs (NEB) LAMP primer design tool^5^. The free energy (ΔG) of selected primers was less than –4 kcal/mol, as a parameter chosen based on oligo stability (Parida et al., 2008). The set of primers used in this study is listed in Table 3 and additional information can be found in Figure 9 and Supplementary Figures 4–6. We designed and validated different LAMP primer sets, such as *N* gene Set1 and Set2 that appeared in other independent researches (Figure 9 and Table 3). *N*2 and *E*1 primer sets were previously designed by Zhang et al. (2020b). The oligos were purchased from Integrated DNA technologies (IDT, Coralville, IA, United States) and from Exxtend (Paulínia, SP, Brazil). All oligos were synthesized at 25 nanomole scale and purified by standard desalting. Thermodynamic evaluation of primers targeting SARS-CoV-2 *N*, *E*, and *RdRp* genes was performed as previously described (Miranda and Weber, 2021). Briefly, hybridization temperature of F3, FIP (F1c+F2), BIP (B1c+B2), LF, and LB primer sets were calculated upon aligning to SARS-CoV or other coronavirus (non-SARS) genomes, considering potential mismatches. The SARS-CoV-2 coverage for each primer was also obtained (Supplementary Table 1).

**TABLE 3:** Sets of LAMP oligonucleotides used in this study.

LAMP primer	Sequence (5′–3′)	References
N_Set1_F3	TGGCTACTACCGAAGAGCT	Ben-Assa et al., 2020; Bhadra et al., 2021; Song et al., 2021; Rabe and Cepko, 2020; Thi et al., 2020; Zhang et al., 2020a; Anahtar et al., 2021; this study
N_Set1_B3	TGCAGCATTGTTAGCAGGAT	
N_Set1_FIP	TCTGGCCCAGTTCCTAGGTA GTGACGAATTCGTGGTGGTGA	
N_Set1_BIP	AGACGGCATCATATGGGTTGC ACGGGTGCCAATGTGATCT	
N_Set1_LF	TGGACTGAGATCTTTCATTTTACCG	
N_Set1_LB	ACTGAGGGAGCCTTGAATACA	
N_Set2_F3	TGGACCCCAAAATCAGCG	Huang et al., 2020; González-González et al., 2021; this study
N_Set2_B3	GCCTTGTCCTCGAGGGAAT	
N_Set2_FIP	CCACTGCGTTCTCCATTCTGGTAA ATGCACCCCGCATTACG	
N_Set2_BIP	CGCGATCAAAACAACGTCGGCCC TTGCCATGTTGAGTGAGA	
N_Set2_LF	TTGAATCTGAGGGTCCACCAAA	
N_Set2_LB	GGTTTACCCAATAATACTGCGTCTT	
E_Set1_F3	TGATGAGCCTGAAGAACATG	This study
E_Set1_B3	CGCTATTAACTATTAACGTACCT	
E_Set1_FIP	TCGGTTCATCATAAATTGGTTCCAT CAAATTCACACAATCGACGG	
E_Set1_BIP	ACGACTACTAGCGTGCCTTTGTCT CTTCCGAAACGAATG	
E_Set1_LF	ACTGGATTAACAACTCCGGATGA	
E_Set1_LB	GTAAGCACAAGCTGATGAGTACGAA	
RdRp_F3	CTGTCAAATTACAGAATAATGAGC	This study
RdRp_B3	TCCATCACTCTTAGGGAATC	
RdRp_FIP	TGTCATCAGTGCAAGCAGTTTGCTG TTGCACTACGACAGA	
RdRp_BIP	ATGCGTTAGCTTACTACAACACACC CATTTCAAATCCTGTAAATCG	
RdRp_LF	ACCGGCAGCACAAGACA	
RdRp_LB	ACAAAGGGAGGTAGGTTTGTACT	
N2_F3	ACCAGGAACTAATCAGACAAG	Butler et al., 2020; Zhang et al., 2020b
N2_B3	GACTTGATCTTTGAAATTTGGATCT	
N2_FIP	TTCCGAAGAACGCTGAAGCGGAAC TGATTACAAACATTGGCC	
N2_BIP	CGCATTGGCATGGAAGTCACAATTT GATGGCACCTGTGTA	
N2_LF	GGGGGCAAATTGTGCAATTTG	
N2_LB	CTTCGGGAACGTGGTTGACC	
E1_F3	TGAGTACGAACTTATGTACTCAT	Butler et al., 2020; Zhang et al., 2020b
E1_B3	TTCAGATTTTTAACACGAGAGT	
E1_FIP	ACCACGAAAGCAAGAAAAAGAAG TTCGTTTCGGAAGAGACAG	
E1_BIP	TTGCTAGTTACACTAGCCATCCTTA GGTTTTACAAGACTCACGT	
E1_LF	CGCTATTAACTATTAACG	
E1_LB	GCGCTTCGATTGTGTGCGT	

*Ref, references where the DNA oligos where originally published or share the same set of primers; F3/B3, outer forward (F) and backward primers; FIP/BIP, inner primers; LF/LB, loop primers. For detailed information on targeted SARS-CoV-2 sequence used, refer to Supplementary Figures 4–6 and Figure 9.*

### Reverse Transcription Loop-Mediated Isothermal Amplification Assays

All mix preparations for RT-LAMP reaction were performed on ice inside a biosafety level 2 cabinet. RT-LAMP reactions were performed according to NEB recommendations, containing the following components: 10 μL of WarmStart^®^ Colorimetric LAMP 2× Master Mix [NEB #M1800 or #M1804, the latter contains dUTP UDG (uracil-DNA-glycosylase) to avoid carryover contamination; composition of both are NEB’s proprietary]—ready-to-use mixture of WarmStart^®^ *Bst* 2.0 DNA polymerase and WarmStart^®^ RTx (reverse transcriptase for one-step transcription/amplification reaction) in presence of a pH sensor that turns from fuchsia (pink) to yellow in presence of increased proton (acid pH) during DNA polymerization on isothermal amplification, 1.6 μmol/L forward inner/backward inner primers (FIP/BIP); 0.2 μmol/L forward and backward outer primers (F3/B3), and 0.4 μmol/L loop forward and loop backward primers (LF/LB); Ultra-pure^TM^ DNAse/RNase-free distilled water (Invitrogen^TM^ #10977015) was added in quantity enough to complete the final volume reaction of 20 μL; isothermal amplification was performed on Veriti^TM^ thermal cycler (Applied Biosystems, Foster City, CA, United States) at 65°C for 30 min. From clinical samples in the first batch, we used as input, 1 μL of RNA extracted from nasopharyngeal swab placed on guanidine-containing VTM, whereas upon optimization, 5 μL source template was considered from the samples in the second group.

When using raw RNA extraction–free samples, we initially prepared a 1:10 ultrapure water diluted clinical sample (1 μL of VTM sample in 9 μL water) and used 1 μL as RT-LAMP reaction input. A similar strategy was applied to SARS-CoV-2 VOC/VOI samples. Positive controls were performed either by RNA extraction from Vero E6-derived inactivated SARS-CoV-2, using synthetic SARS-CoV-2 *N* gene-harboring plasmid (ECRA Biotech, Campinas, SP, Brazil #EB14-20) or inactivated laboratory-cultured SARS-CoV-2, when aiming the RNA extraction–free tests. For optimization purposes, incubation time tested varied from 30 to 50 min. The first 100 RT-LAMP reaction products were migrated in 2% agarose gel to confirm specific amplification in positive reactions and amplicon-free nontemplate controls. Gel images were taken using the ImageQuant^TM^ LAS 4000 with GelRed^TM^ (Biotium #41003) as intercalating dye. Non–SARS-CoV-2 RNA extracted samples of influenza A, influenza B, hRSV, dengue, Zika, Chikungunya, and yellow fever viruses were also added as 1-μL input.

### Analytical Sensitivity

Absolute quantification was performed based on a calibration curve prepared using the standard SARS-CoV-2 *E* gene–harboring plasmid (2 × 10^5^ copies/μL; Biogene COVID-19 PCR, Bioclin/Quibasa #K228-1; Lot: 0007), SARS-CoV-2 (2019-nCoV) Charité/Berlin primer probe panel (IDT, #10006804), and the GoTaq^®^ Probe 1-step RT-qPCR System (Promega #A6120), according to manufacturer instructions, as indicated by the US CDC. Real-time RT-PCR program was performed as follows: first stage (×1) 15 min at 45°C, second stage (×1) 2 min at 95°C, and third stage (×40) 3 s at 95°C followed by 30 s at 55°C. Linear regression was performed using Prism software, version 9 (GraphPad Software, San Diego, CA, United States) leading to the equation: *Y* = –3.6383*X* + 38.771 and coefficient of correlation *R*^2^ = 0.9938 (Supplementary Figure 3). Viral RNA either from Vero E6-derived SARS-CoV-2 (SARS-CoV-2 isolate HIAE-02: SARS-CoV2/SP02/human/2020/BRA GenBank accession no. MT126808.1) or obtained from clinical nasopharyngeal swabs was quantitated based on the Ct value for E gene.

## Data Availability Statement

The datasets presented in this study can be found in online repositories. The names of the repository/repositories and accession number(s) can be found below: https://www.gisaid.org/, The genomes of SARS-CoV-2 VOI and VOCs generated are deposited on GISAID according to the following accession codes: EPI_ISL_2221860, EPI_ISL_2221850, EPI_ISL_2221873, EPI_ISL_2221890, EPI_ISL_2221902, EPI_ISL_2221885, EPI_ISL_2221844, and EPI_ISL_2221866.

## Ethics Statement

The studies involving human participants were reviewed and approved by Research Ethics Committee involving human beings at Instituto René Rachou, Fundação Oswaldo Cruz, under license protocol number: 4084902 and CAAE (certificate of presentation for ethical appreciation): 31984720300005091. The ethics approval was issued on June 12, 2020. Written informed consent for participation was not required for this study in accordance with the national legislation and the institutional requirements.

## Author Contributions

PA, IB, and RM-N: conceptualization and experimental design. EO, AF-L, LA, AG, IB, and FR: investigation and performed experiments. AF-L, LA, AG, EO, PA, and RM-N: analyzed the data. MB, PM, FC, HM, GW, ST, and GLW: contributed to reagents, materials, and analysis and tools. PA and RM-N: supervision. RM-N, EO, AF-L, and RR: writing—original draft. PA, ST, GLW, GW, MB, and RM-N: writing, review, and editing. All authors discussed the results and contributed to the final manuscript.

## Conflict of Interest

HM is part of Visuri company. Results presented here are the basis of a COVID-19 RT-LAMP diagnostic test offered by Visuri named OmniLAMP^®^ SARS-CoV-2 kit. PA and RM-N are co-founders and scientific advisors at CEPHA Biotech. The remaining authors declare that the research was conducted in the absence of any commercial or financial relationships that could be construed as a potential conflict of interest.

## Publisher’s Note

All claims expressed in this article are solely those of the authors and do not necessarily represent those of their affiliated organizations, or those of the publisher, the editors and the reviewers. Any product that may be evaluated in this article, or claim that may be made by its manufacturer, is not guaranteed or endorsed by the publisher.

## Footnotes

1
https://coronavirus.jhu.edu/

2
https://www.worldometers.info/coronavirus

3
https://cov-lineages.org

4
https://primerexplorer.jp/e/

5
https://lamp.neb.com
